# Genotypic tropism testing by massively parallel sequencing: qualitative and quantitative analysis

**DOI:** 10.1186/1472-6947-11-30

**Published:** 2011-05-13

**Authors:** Martin Däumer, Rolf Kaiser, Rolf Klein, Thomas Lengauer, Bernhard Thiele, Alexander Thielen

**Affiliations:** 1 Institute of Immunology and Genetics, Pfaffplatz 10, 67655 Kaiserslautern, Germany; 2 Institute of Virology, University of Cologne, Fürst-Pückler-Str. 56, 50935 Cologne, Germany; 3 Max Planck Institute for Informatics, Stuhlsatzenhausweg E1.4, 66123, Saarbrücken, Germany

## Abstract

**Background:**

Inferring viral tropism from genotype is a fast and inexpensive alternative to phenotypic testing. While being highly predictive when performed on clonal samples, sensitivity of predicting CXCR4-using (X4) variants drops substantially in clinical isolates. This is mainly attributed to minor variants not detected by standard bulk-sequencing. Massively parallel sequencing (MPS) detects single clones thereby being much more sensitive. Using this technology we wanted to improve genotypic prediction of coreceptor usage.

**Methods:**

Plasma samples from 55 antiretroviral-treated patients tested for coreceptor usage with the Monogram Trofile Assay were sequenced with standard population-based approaches. Fourteen of these samples were selected for further analysis with MPS. Tropism was predicted from each sequence with geno2pheno_[coreceptor]_.

**Results:**

Prediction based on bulk-sequencing yielded 59.1% sensitivity and 90.9% specificity compared to the trofile assay. With MPS, 7600 reads were generated on average per isolate. Minorities of sequences with high confidence in CXCR4-usage were found in all samples, irrespective of phenotype. When using the default false-positive-rate of geno2pheno_[coreceptor] _(10%), and defining a minority cutoff of 5%, the results were concordant in all but one isolate.

**Conclusions:**

The combination of MPS and coreceptor usage prediction results in a fast and accurate alternative to phenotypic assays. The detection of X4-viruses in all isolates suggests that coreceptor usage as well as fitness of minorities is important for therapy outcome. The high sensitivity of this technology in combination with a quantitative description of the viral population may allow implementing meaningful cutoffs for predicting response to CCR5-antagonists in the presence of X4-minorities.

## Background

Treatment of HIV infection has progressed significantly in the last decade. Amongst other factors this improvement is also based on the development of new drugs which are becoming more and more potent and which nowadays are given in combination [1]. This so-called highly active antiretroviral therapy (HAART) aims at inhibiting the viral replication as strongly as possible by using antiretroviral drugs from usually two different drug classes. While mortality and morbidity decreased substantially, long-term side effects and suboptimal drug potency are still major obstacles [2]. Moreover, the emergence of drug-resistant variants from minor populations may lead to early therapy-failures despite apparently effective treatment regimes [3].

With the approval of maraviroc (MVC, Celsentry/Selzentry, Pfizer) [4] in 2007, a further new class of antiretrovirals was introduced into anti-HIV treatment. In contrast to previously approved drugs, drugs from this class do not bind to viral proteins but to a specific coreceptor which is expressed by the host cell [5,6]. Two coreceptors CCR5 and CXCR4 have been shown to be relevant in vivo. The mode of action of coreceptor antagonists led to the assumption that administration of such drugs would impede the adaptation of the virus and consequently lower the risk of developing resistance. However, the major problem of coreceptor antagonists is that there are two types of viruses: 1) so-called R5-viruses which use the CCR5 coreceptor for cell entry and which are usually dominant in the beginning of infection, and 2) dual-tropic and X4-viruses, which can also use another coreceptor (CXCR4) to gain entry into cells. Due to the fact, that maraviroc binds to CCR5, viruses which are capable of using CXCR4 are not harmed by the drug. Therefore, coreceptor usage, also known as viral tropism, has to be determined before administration of a regimen containing maraviroc. Currently such tests are performed with a phenotypic assay, usually the trofile assay (nowadays the enhanced sensitivity version) from Monogram Biosciences (San Francisco, CA, USA) [7]. In this assay a recombinant replication-defective virus carrying the tropism-determining gp120 V3 region of a clinical isolate, is analyzed for entry on either CCR5- or CXCR4 expressing cells. The assay has been used in all maraviroc and vicriviroc (another coreceptor antagonist which is not in development anymore, Merck) clinical trials and has therefore become the *de facto *gold standard for measuring coreceptor-tropism. The main disadvantages of phenotypic assays are that they are very time-consuming and cost-intensive [8]. Additionally, samples with viral loads below 1,000 copies/ml or certain non-B subtypes exhibited an extraordinarily high failure rate in the original Trofile assay.

Here the genotypic approach poses an alternative. It is characterized by the experimental determination and computational interpretation of the viral genome. Genotypic prediction of HIV-1 tropism is an inexpensive and fast alternative to phenotypic assays [8,9]. However, standard sequencing approaches afford only a low sensitivity of X4-detection, especially in clinical isolates [10-12]. They generally work well when applied to clonal data [10,12] which led to the conclusion that "false" predictions are mainly attributed to the lack of detection of minor populations of CXCR-using variants. To overcome this disadvantage, a new approach to genotypic tropism testing, the so-called massively parallel sequencing [13] was introduced. This technology enables generating an unprecedented number of sequences on the basis of single molecule sequencing, thus increasing the probability of identifying minority variants in heterogeneous gene families or virus populations. Therefore, coreceptor usage prediction from genotype is generally regarded as a promising application of ultra-deep sequencing, which detects single clones, thereby being much more sensitive than classical population-based Sanger sequencing.

In this study we address the question whether massively parallel sequencing can be successfully combined with bioinformatic approaches in order to afford improved qualitative and a quantitative prediction of coreceptor usage from the V3-loop.

## Methods

### Patients

Samples from 55 heavily pre-treated patients with limited therapy options were screened for potential administration of maraviroc. 3 ml plasma from each patient was shipped on dry ice for phenotypic tropism testing (original trofile tropism assay, Monogram Biosciences, South San Francisco). Plasma viral load was determined using the M2000 system (Abbott Molecular). Results were documented as either CCR5-tropic (R5), CXCR4-tropic (X4) or dual-/mixed-tropic (D/M). X4- and D/M isolates are pooled together and called X4 from here on. The investigation was performed within the German RESINA-study for which the patients signed an informed consent (BMG 310/4476/02/3).

### RNA extraction, cDNA synthesis and V3-PCR

RNA extraction was performed by using the Viral RNA mini kit (Qiagen, Hilden, Germany) according to the manufacturer's instructions. cDNA-synthesis was performed using 10 μl of RNA, specific primers V3-1 (5'TACAATGTACACATGGAATT, position 6958 → 6977 in HXB2), V3-2 (ATTACAGTAGAAAAATTCCCC, position 7362 → 7382 in HXB2) [14] and Superscript II (Invitrogen) according to the manufacturer's instructions in a total volume of 20 μl.

Amplification of the V3 region was carried out using the FastStart HiFi PCR system, (Roche, Mannheim) and primers V3-for (TGGCAGTCTAGCAGAAGAAG, position 7010 → 7029 in HXB2) and V3-rev (CTGGGTCCCCTCCTGAGG, position 7315 → 7332 in HXB2) [14] in a Primus 96 plus thermal cycler (MWG-Biotech).

Products to be massively parallel sequenced were generated with fusion primers V3FusA: GCCTCCCTCGCGCCATCAG-V3-for and V3FusB: GCCTTGCCAGCCCGCTCAG-V3-rev.

### Standard DNA sequencing and massively parallel pyrosequencing

Clean-up of the PCR products prior to standard sequencing was performed by incubation with FastAP™ in conjunction with Exonuclease I (Fermentas, Burlington, Canada) for 10 min at 37°C followed by heat inactivation for 5 min at 75°C.

Population-based sequencing of the V3 region was carried out using the Big Dye^® ^Terminator v3.1 cycle sequencing kit (Applied Biosystems, Foster City, CA, USA) with primers (3pmol) V3-for and V3-rev. Extension products were purified using the Biomek NXp automated sequencing reaction cleanup system (Beckman Coulter, Fullerton, CA, USA) and were run on an ABI 3130 capillary sequencer (Applied Biosystems, Foster City, CA, USA).

Out of the 55 samples, 14 (7 R5, 7 X4) were randomly selected for further analysis with massively parallel sequencing. All 14 amplicons were purified with AMPure magnetic beads (Agencourt, Beckman Coulter) and quantified using an Agilent 2100 Bioanalyser (Agilent Life Sciences). Since bidirectional sequencing using primers A and B might result in unbalanced read composition, unidirectional sequencing with primer A (kit II; Roche-454 Life Sciences) was chosen to ensure precise quantification. After beads recovery and enrichment, approximately forty thousand beads were loaded on each region of a GS FLX PicoTiter plate subdivided with a 16-lane gasket. Sequencing was performed on a Genome Sequencer FLX (Roche-454 Life Sciences).

### Determination of HIV-coreceptor phenotype

The trofile assay is a single-cycle recombinant virus assay developed by Monogram Biosciences. A 2.5kb long part of the patient-derived *env *gene is amplified by PCR and inserted into an envelope expression vector. Together with a replication-defective retroviral vector carrying a luciferase reporter gene, this vector is used to co-transfect human embryonic kidney 293 cells (HEK293). Pseudo-viruses produced by these cells are then given to engineered U87 target cell lines expressing either CCR5 or CXCR4.

Upon successful infection of these cells, the reporter gene is expressed and a light signal emitted. This can be quantitated in relative light units (RLU) and is additionally controlled by the presence of coreceptor antagonists. The original tropism assay is reported to be reproducible and effective at detecting minority populations of CXCR4-using virus at levels as low as 5% [7].

### Preprocessing of 454-data

Massively parallel sequencing data was processed directly from the Standard Flowgram Files (.sff) containing the sequence trace data. The system's methods for post-processing (GS De Novo Assembler, GS Reference Mapper, GS Amplicon Variant Analyzer) have not been used due to previous problems with this software

Two main filtering steps were applied to minimize possible errors and to sort out non-V3 sequences arising from PCR- or sequencing errors. These worked as follows: First, sequences were translated into all three reading frames. Each frame was then screened for typical start and end-motifs of the V3-loop (e.g. CTR, CIR, AHC, AYC). Only sequences where both ends of the loop were recognized in the same reading frame were further processed. The potential V3-loop was cut out and its length analyzed. While the usual V3-loop has a length of about 35 amino acids, loops containing a number of insertions and deletions have been observed previously, as well [11]. To allow for such indels but limit the number of deviations from the standard loop which could occur due to sequencing problems, we introduced the criteria that V3-loops had to have a length of at least 30 and at most 45 residues. If several V3-start- or V3-end motifs were found in a specific read, only the one with the length closest to 35 was selected for coreceptor usage prediction.

### Tropism prediction from genotype

Genotypes resulting from both sequencing methods were input to a development version of geno2pheno[coreceptor] for coreceptor usage prediction (version from June 2009) [11]. This version of the freely available web system produces exactly the same predictions as the public version but differs in two respects: First, it can process a number of sequences in batch allowing to handle the vast amount of data generated with the Genome Sequencer FLX system and second it returns the internal scores of the prediction method which are currently not displayed on the website (the displayed FPR-scores are a transformation of these scores ).

Prediction results were used in two fashions: On the one hand, cutoffs corresponding to the false-positive rates available on the website (1%, 2.5%, 5%, 10%, 15%, 20%) were used in order to classify the samples into R5- and X4-viruses. On the other hand, the raw values were used for detailed quantitative analysis as described below.

### Performance assessment

For performance comparison, the Monogram Trofile assay was used as reference. Performance was measured in terms of accuracy, true positive rate and false positive rate. The true positive rate is the relative number of X4 viruses correctly predicted as X4 while the false positive rate is the relative number of R5 viruses falsely classified as X4. Accuracy represents the total agreement between genotype and phenotype. For all evaluations, the statistical programming language *R *[15] was used.

### Analysis of the error rate

The 454 GS-20 system is reported to have an overall error rate of 0.4% [16] to 0.49% [17] whereas the error rate of the FLX system is supposed be about 0.12% [16]. For the GS-20 system, only about 11% [16] to 16% [17] of all errors turned out to be substitution errors whereas the remaining errors are insertion and deletion errors. Thus, the per-base substitution error rate is approximately 0.042% [16] to 0.078% [17]. A nucleotide error might not necessarily change the amino acid of the respective codon and thus the prediction score. When checking the number of possible synonymous and non-synonymous mutations, we found 460 of 576 possible codon mutations to be non-synonymous. Therefore, the probability of a problematic substitution error in these reports is reduced to 0.03354% and 0.0623%, respectively. Given a V3-loop of 105 nucleotides, the probability of having no error is therefore (1-0.0335%)^105 ^= 96.54% and (1-0.0623%)^105 ^= 93.66% and consequently the probability of an erroneous V3 amino acid sequence only 3.4% to 6.3%. Considering the fact that not every amino acid change will change the coreceptor prediction we assume that such errors do not affect our results substantially.

Insertion and deletion errors can also be considered to be unproblematic because they induce frame shifts. These are detected by our filtering approach testing for valid V3-ends at both sides of the loops. Last but not least, it might happen that within a V3-loop an insertion and a deletion error occur together so that the V3-ends are in the same frame. However, reads with more than one sequence error occurred only in 7% of the reads in [17]. Taken into account that both errors have to be located within the V3-loop which is much smaller than the whole read and one of them has to be an insertion and one a deletion error the probability of such a case is negligibly small, too.

Based on these considerations we can assume that our results should not be affected too much by problems with the sequencing technology.

## Results

### Bulk-sequencing results

Prediction results based on population-sequenced isolates were compared with tropism calls generated by the trofile assay (see Table 1). Using the default false-positive rate of geno2pheno (10%), 43 of the 55 isolates were predicted concordantly with the Monogram Trofile Tropism Assay. For 9 out of 22 samples phenotyped to be X4 by the trofile assay, geno2pheno_[coreceptor] _predicted an R5-virus. On the other hand, for 3 of the 33 viruses determined by the trofile assay as R5, geno2pheno returned an X4-prediction. Hence, the sensitivity of predicting CXCR4-using isolates was 59.1% while specificity was at 90.9%. These prediction results are in line with results reported in recent publications [10,11,18-20]. The overall accuracy with respect to the trofile assay was 78.2%.

**Table 1:** Comparison of genotype and phenotype

	geno2pheno[coreceptor]	
	R5	X4

Trofile-R5	30	3

Trofile-X4	9	13

Comparison of trofile-results and geno2pheno predictions from bulk-sequenced isolates tested for maraviroc administration.

### 454-results

#### General statistics

Table 2 shows characteristics of the individual samples analyzed with the GS FLX system. On average, around 7600 (range: 885-12992) reads were generated per isolate. The variation in the number of generated reads per sample remained unclear. It was not significantly correlated with the viral load (r^2 ^= 0.042). The mean viral load was at 35810 copies per milliliter.

**Table 2:** Properties of analyzed patient isolates

isolate	trofile	viral load, copies/ml	#reads	#reads with v3 loop	#variants
1	X4	65815	885	667	67

2	X4	3236	4614	3205	233

3	X4	28850	3875	2825	210

4	X4	199005	12063	10091	325

5	X4	10082	12013	9608	584

6	X4	9400	3094	1581	147

7	X4	48354	7847	4833	388

8	R5	48000	10622	8621	354

9	R5	9300	1601	344	57

10	R5	16400	6105	4905	216

11	R5	11928	11142	8677	301

12	R5	1270	11551	8748	283

13	R5	18709	12992	10555	320

14	R5	31000	7740	5808	441

Characteristics of the isolates sequenced with the 454-machine. The number of variants is the number of different amino acid sequences found for the v3 loop.

After translation and adjusting for the V3-loop we found 280 different amino acid variants of the V3-loop on average (range: 57-584). The squared correlation coefficient between the number of different V3-variants and the number of reads was r^2 ^= 0.584 (p = 0.001). When comparing R5- and X4 isolates, we did not find any significant difference between the two groups neither for the number of reads generated (p = 0.29, 8821 vs. 6341), nor for the viral load (p = 0.26, 19515 vs. 52106) or the number of found V3-loops (p = 0.29, 6808 vs. 4687) and their variants (p = 0.97, 281.7 vs. 279.1).

#### Quantitative analysis of coreceptor usage

**Figure 1:**
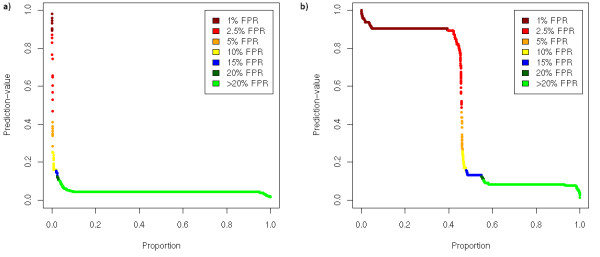
**Overview of coreceptor usage predictions from one sample (a: isolate 13, Trofile R5; b: isolate 5, Trofile X4)**. Every dot represents the prediction for one read. The colors indicate at which FPR-cutoff, the read would have been predicted as X4.

All reads in which a consistent V3-loop sequence was found were input to the geno2pheno_[coreceptor] _prediction system. This resulted in around 7,600 coreceptor predictions per isolate. Since this vast amount of data cannot be interpreted easily we decided to display the results in a plot in addition to tabulating numbers. Figure 1 shows the results for two isolates. Every individual point in these plots represents the prediction score of one read. The score of the prediction which can be regarded as an affinity value is plotted along the y-axis. High scores represent predictions for which geno2pheno_[coreceptor] _was very confident of the virus being X4 (low false-positive rates) whereas low scores represent viruses probably being R5. The shape of the curve qualitatively reflects the coreceptor usage of the whole population within the relevant isolate. E.g. the population depicted in Figure 1a consists of almost exclusively R5-viruses whereas Figure 1b depicts a virus population about half of which can use the CXCR4-receptor.

**Figure 2:**
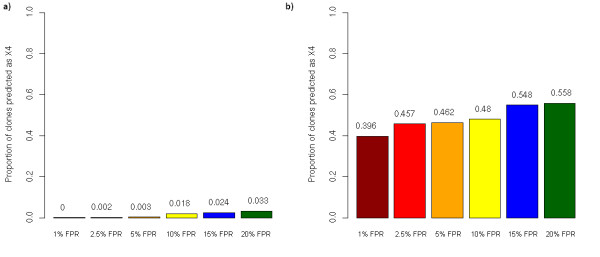
**Proportion of predicted X4-viruses at different FPR in ultra deep sequenced V3 amplicons (a: isolate 13 Trofile R5; b: isolate 5, Trofile X4)**.

In addition, we generated histograms displaying the frequency of predicted X4-viruses for the specific false-positive rates on the geno2pheno website (see Figure 2). These are useful when one is interested in the question how many viruses are predicted to be X4 at a specific cutoff. While being more easily to interpret than the previous type of graph, the global picture of the viral population is lost. E.g. from Figure 1b one can easily infer that the viral population is split into two major strains while this is not possible from Figure 2b.

#### Comparison between genotypic prediction and phenotypic results

In order to obtain a qualitative prediction from the ultra-deep data, two cutoffs have to be defined. The first is a cutoff for the prediction score has to be selected - reflecting the affinity of the virus to the CXCR4-coreceptor. For this purpose and in order to be comparable to the bulk-sequenced isolates, we chose the cutoff resulting in the standard 10% false-positive rate of geno2pheno_[coreceptor]_. Second, we have to set a minority threshold because in every sample individual clones very predictive for CXCR4-usage had been found. Three reasons might be responsible for this: (i) not every minority is clinically relevant, (ii) the phenotypic assay also has a sensitivity limit which has to be taken into account and which is most probably higher than the limit for massively parallel sequencing, and (iii) in some cases the 454-machine also produces erroneous sequences that are not recognized during preprocessing and prediction and which should not have an impact on the overall prediction outcome. Hence, we decided to select a minority cutoff of 5%, i.e. only samples in which more than 5% of the sequences were predicted to be CXCR4-using were assigned to be X4. This value is identical with the proposed sensitivity limit of the standard trofile assay used in this analysis.

By applying these settings to the dataset, we could achieve concordance with the phenotypic assay in all but one case (isolate 7, see Table 3).

**Table 3:** Tropism results from different assays

*isolate*	*phenotype *(trofile)	*prediction from bulk-sequence, predicted as X4 at FPR*^*a *^*of:*	*MPS, proportion. predicted as X4*^*b*^
1	X4	2.5%	85.3%

2	X4	5%	87.0%

3	X4	10%	14.2%

4	X4	15%	5.4%

5	X4	15%	48.0%

6	X4	---^c^	68.5%

7	X4	---	1.0%

8	R5	10%	1.4%

9	R5	---	3.4%

10	R5	---	0.3%

11	R5	---	0.5%

12	R5	---	1.6%

13	R5	---	1.8%

14	R5	---	2.4%

Tropism results from different approaches used: phenotype (trofile), coreceptor usage prediction from bulk-genotype, and coreceptor usage prediction from massively parallel sequencing.

FPR: false-positive-rate of geno2pheno.

^a^the displayed value is the highest cutoff at which an isolate would have been predicted as X4

^b^default geno2pheno[coreceptor]-cutoff of 10% FPR used

^c ^'---' if predicted FPR, was above the highest cutoff tested (20%)

## Discussion

In this work, we analyzed the usefulness of next-generation sequencing technologies in the realm of coreceptor usage prediction. Our results show that the approach affords precise classification as well as the possibility of quantitatively assessing the tropism of the viral quasispecies. With respect to the classification, our results are concordant with the phenotype in all cases but one. It should be noted that the cutoffs selected in this work were not optimized. Instead we selected cutoffs which are plausible (default cutoff of the geno2pheno method and the proposed sensitivity limit of the phenotypic assay). On the other hand, due to the limited amount of data, it was not possible to infer the cutoffs automatically in a statistically sound way. Future work on larger datasets should focus on optimizing the cutoffs. The data set on which we base this study is too small for this purpose. However, when comparing the results to the predictions obtained with conventional bulk-sequencing approaches it could clearly be shown that combining genotypic prediction with ultra-deep sequencing provides a fast and accurate alternative to phenotypic assays.

In addition to simple classification of the isolates into R5- and X4-samples, we could also provide a quantitative output with this approach. This may be of minor interest when comparing against a phenotype as done in this work but can be more important in the context of administration of coreceptor antagonists. A patient with a very small minority of X4-viruses might respond differently to a regimen containing coreceptor antagonist than a patient with a majority of X4-viruses despite the fact that both might have an X4-phenotype. However, this has to be addressed in the future when larger datasets with therapy outcome become widely available.

The main advantage of the massively parallel sequencing technology implemented in the GS FLX-system is that it can reliably detect minor variants and generate clonal sequences. In the context of coreceptor usage, especially the first property seems to be of special interest because minor variants have been shown to be much more important in tropism determination than in HIV resistance testing.

The main disadvantage of the 454-technology is that currently it is rather expensive with costs for a run ranging around 10,000€. However, several samples can be sequenced in parallel in a single run so that it is possible to lower the per/sample price dramatically (e.g. by sequencing 48 patients in parallel the price will decrease to about 200€). The drawback is that usually not that many patients have to be screened at the same time. Nevertheless, sequencing prices will further decrease so that we believe that this technology will be standard in the future.

In the course of this work, some publications have become available dealing with coreceptor usage and massively parallel sequencing. Tsibris et al. [21] have used the 454-technology to monitor the evolution and escape of HIV-1 during CCR5 antagonist therapy in four patients but not to predict the tropism of these viruses. In a related work, Archer et al. [22] used the 454 Genome Sequencer 20 to find minorities of CXCR4-using variants emerging in a patient treated with maraviroc monotherapy. In contrast to our work, however, these two works did not focus on the prediction of viral tropism but on the observation of shifts within the viral quasispecies under selective drug pressure. More recently, approaches similar to the one discussed here have been presented by Abbate et al. [23] and Vandekerckhove et al. [24]. Swenson et al. used such a method to rescreen the patients enrolled in the MOTIVATE studies and compared the results with the ones generated by the original trofile assay [25,26]. In a related study it was also found that genotypic interpretation of data from population-based sequencing works much better than anticipated despite substantial discordant rates to the original trofile assay [27].

The original trofile assay used in this work has been replaced by an enhanced version (ESTA) able to detect minor variants circulating in quantities as low as 0.3%. This might imply that more differences with the new assay would have been seen than with the old assay. However, we believe that this is not the case for two reasons: First of all, we have set the minority cutoff to 5% to mirror the one of the original trofile assay which was reported to be 100% sensitive at detecting 10% minor variants and 85% sensitive at detecting 5% minor variants in a mixed virus population. We could easily lower this cutoff to any other value. Second, we have presented data at the 7^th ^European HIV Drug Resistance Workshop [28] on how to adjust the FPR cutoff of geno2pheno[coreceptor] in order to obtain prediction results comparable in terms of sensitivity and specificity of X4-detection on samples generated with the original and the enhanced sensitivity trofile assays. The same adjustment could be applied in this case as well.

Additional data would definitively strengthen the results. However, we cannot afford the high costs to screen further samples with the Trofile assay and with the 454-technology. Moreover, we would now get the results from the enhanced sensitivity Trofile and not the original Trofile used in this study. Thus, we would also have to rescreen the 14 samples used in this analysis again.

There is room for improvements. First, as already discussed above one has to decide if the goal is to recreate the results of Trofile-assay or rather to reliably predict coreceptor antagonist admissibility. In our opinion the latter is more important. Therefore the 5%-minority cutoff chosen in this project is probably not the best solution and should be adjusted when more treatment data become available. In fact, the 5% cutoff appears high with respect to the possibilities of ultradeep sequencing. We chose it since we compare the 454 technology against an assay for which a minority threshold of about 5% is suitable. If we used a cutoff of 1%, the samples 8 and 12-14 would also be predicted as X4 by massively parallel sequencing but not by the Trofile assay. We could then claim that the 454 assay is more sensitive than the Trofile assay. Others might say, however, that these samples are erroneously classified as X4 on the basis of the 454 measurements. Thus, we feel that a fair comparison is only possible if we use the same sensitivity limits for both assays compared.

Furthermore, one might think about changing the dogma to exclude all patients from a therapy with coreceptor antagonists who harbor a dual-tropic or X4-virus. We found at least one sequence predicted as X4 in every isolate which means that if we do not use a minority cutoff none of our patients would be administered a coreceptor antagonist. This does not make therapeutic sense. Instead of trying to further increase the sensitivity of detecting minority populations we suggest to search for clinically relevant cutoffs. These cutoffs might not only be based on the number of minor X4-viruses but also depend on the fitness of these variants. McGovern et al. [27] determined and proposed a clinical cutoff at FPR of 5.75%. However, this cutoff was based on triplicate sequencing in which the lowest predicted FPR was used. Consequently, these FPRs were lower in general than those for single sequencing making a direct comparison difficult. We feel that with respect to the phenotype, the standard 10% g2p cutoff is more appropriate.

Another point one to be addressed in the future is that we used a standard coreceptor prediction tool which was originally developed for bulk-sequenced isolates and not for ultra-deep data. This might imply problems because e.g. the prediction system also uses features like the number of ambiguous positions which are usually not or only rarely existent in massively parallel sequencing data. Thus, a tool trained specifically on the latter kind of sequence data should show enhanced prediction outcomes.

Finally, coreceptor antagonists are administered together with other antiretroviral compounds. We do not know if minorities of X4-viruses accumulate the same resistance mutations to other drugs as the predominant R5-viruses. There might be the possibility that these variants have quite different polymerase-genes and therefore are resistant against a coreceptor antagonist but not against the other drugs in the backbone. Hence, a future goal is to sequence the whole virus with the ultra-deep sequencing technology in order to address this problem.

## Conclusions

Massively parallel sequencing can be successfully combined with prediction of HIV-1 coreceptor usage from V3 genotype. Such an approach results in a fast and accurate alternative to phenotypic assays which not only gives a qualitative tropism result but also allows for generating a quantitative picture of the whole viral quasispecies.

## Competing interests

The authors declare that they have no competing interests.

## Authors' contributions

MD, RKL and BT performed the 454-sequencing experiments. MD and RK provided the samples and sequenced them with standard bulk-sequencing methods. TL and AT carried out the bioinformatics analysis. MD and AT designed the experiments. All authors helped writing the manuscript, read and approved the final manuscript.

## Pre-publication history

The pre-publication history for this paper can be accessed here:

http://www.biomedcentral.com/1472-6947/11/30/prepub
